# Chitosan-*graft*-poly(*N*-vinylcaprolactam) Nanoparticles Containing *Crotalus atrox* Snake Venom: Biological and Physicochemical Characterization †

**DOI:** 10.3390/nano15191538

**Published:** 2025-10-09

**Authors:** Serena Sophia Rudy, Jorge Jimenez-Canale, Jose A. Sarabia-Sainz, Ana María Guzmán Partida, Alexel J. Burgara-Estrella, Erika Silva-Campa, Aracely Angulo Molina, Marcelino Montiel-Herrera, Nelly Flores-Ramírez, Paul Zavala-Rivera, Daniel Fernández-Quiroz

**Affiliations:** 1 School of Life Sciences, University of Applied Sciences and Arts Northwestern Switzerland, Hofacker Strasse 30, 4132 Muttenz, Switzerland; 2 Department of Research in Polymers and Materials, University of Sonora, Hermosillo 83000, Sonora, Mexico; jorgejimzc@gmail.com; 3 Department of Research in Physics, University of Sonora, Hermosillo 83000, Sonora, Mexico; alexel.burgara@unison.mx (A.J.B.-E.); erika.silva@unison.mx (E.S.-C.); aracely.angulo@unison.mx (A.A.M.); 4 Center for Research in Food and Development, Food Science Research Group, Gustavo Enrique Astiazaran Rosas 46, Hermosillo 83304, Sonora, Mexico; gupa@ciad.mx; 5 Department of Chemical-Biological Sciences, University of Sonora, Hermosillo 83000, Sonora, Mexico; 6 Department of Medicine and Health Sciences, University of Sonora, Hermosillo 83000, Sonora, Mexico; marcelino.montiel@unison.mx; 7 Department of Wood Engineering and Technology, Universidad Michoacana de San Nicolás de Hidalgo, Morelia 58030, Michoacán, Mexico; nelly.flores@umich.mx; 8 Department of Chemical Engineering and Metallurgy, University of Sonora, Hermosillo 83000, Sonora, Mexico; paul.zavala@unison.mx

**Keywords:** thermosensitive nanoparticles, *Crotalus atrox* snake venom, physicochemical and biological properties

## Abstract

The development of snake venom-loaded nanobiosystems based on smart biopolymers represents a promising therapeutic approach in several biomedical research fields. Specifically, the western diamondback rattlesnake (*Crotalus atrox*) contains various bioactive peptides and proteins with reported antitumor activity. This research aimed to establish a simplistic, facile and straightforward protocol for preparing chitosan-*g*-poly(*N*-vinylcaprolactam) nanoparticles containing *C. atrox* venom for potential use as a therapeutic nanocarrier against breast carcinoma cell lines. Herein, the physicochemical properties of venom-loaded nanoparticles were evaluated by FTIR, DLS, and SDS-PAGE. Also, the biological properties of both *C. atrox* venom and Cs-Venom NPs such as hemagglutination and hemolysis activity were evaluated in vitro. Finally, we evaluated their cytotoxic activity against two breast carcinoma cell lines (T-47D and MDA-MB-231). The most suitable formulation exhibited a hydrodynamic size of 222 nm, a ζ-potential of 42.0 mV and an encapsulation efficiency of 88.6%. *C. atrox* venom exhibited hemagglutination at concentrations >15 µg/mL but, no hemagglutination or hemolysis was observed for the CS-Venom NPs. Lastly, the IC50 of Cs-Venom NPs was determined for the T-47D and MDA-MB-231 cell lines, at 61.7 and 59.0 µg/mL, respectively. Thus, Cs-Venom NPs exhibit promising properties that can be considered a feasible alternative for developing controlled-release therapeutic systems.

## 1. Introduction

Nowadays, biopolymer-based nanomaterials represent an encouraging platform for performing drug delivery systems. Their suitability as therapeutic agents is based on favorable properties such as biocompatibility, low or no toxicity, as well as their ability to associate with bioactive macromolecules and be released at specific sites in the body [1,2,3]. Stimuli-responsive polymers have garnered attention due to their ability to undergo reversible, nonlinear changes in their local properties in response to environmental stimuli [4,5]. In nanomedicine, studies have focused on variations in pH, temperature, ionic strength, and electric or magnetic fields, among others [5].

In this sense, chitosan-*graft*-poly(*N*-vinyl caprolactam) (Cs-*g*-PVCL) is a temperature-responsive copolymer that exhibits a near-physiological lower critical solution temperature and pH and ionic strength sensitivity [6,7,8]. The cationic nature of their structure confers mucoadhesive properties and can potentiate certain properties for specific applications. Cs-*g*-PVCL has been employed in nanoformulations as a controlled drug delivery system [9] for various anticancer drugs, including curcumin [10], 5-fluorouracil [11], and doxorubicin [12]. These investigations reveal biocompatibility, very low cytotoxicity (hydrolysis does not produce toxic, low-molecular-weight amines), temperature-dependent controlled drug release, and efficacy of these nanoparticles in animal models [13].

Thus, the stimuli-responsive nanoformulations represent an interesting strategy to improve the specificity, biodistribution, and internalization of drugs, thereby reducing side effects in healthy organs. In this sense, cancer is a complex and multifactorial disease that affects a high percentage of the population worldwide. Current treatment strategies for cancer involve invasive procedures, such as radiation, chemotherapy, surgical removal of the damaged tissue, or their combination. However, conventional chemotherapy drug therapies lack specific biodistribution and specificity [14,15]. Nanomedicines have contributed to the development of novel therapeutics that can overcome several key issues, such as low bioavailability and target specificity, which are often reported in currently approved cancer medicines [16]. Novel materials have helped improve this, enhancing their therapeutic properties by avoiding rapid degradation or targeting specific tissue sites [17]. Although biomaterials have significantly improved the development of novel treatments, it is essential to research new molecules to broaden physicians’ options when treating patients. Therefore, researching novel biologically active compounds for cancer therapy represents one of the most promising areas in the fields of biotechnology and pharmaceuticals.

In the last decade, approximately 10% of all FDA-approved drugs have been based on peptides and oligonucleotides (called TIDES). These numbers reinforce the feasibility of using TIDES to treat a variety of conditions, including rare diseases and cancer [18,19]. Interestingly, various animal venoms have been reported to contain highly variable and abundant bioactive peptides and proteins, making them promising resources for potential pharmaceutical ingredients [20]. Mexico has the highest diversity of snakes in the world [21]; some are considered highly venomous (HV) if bitten. Specifically, Sonora has records of more than 10 HV species, and the western diamondback rattlesnake, *Crotalus atrox*, is probably the most common. Different research teams have thoroughly studied their venom [22,23], and many of its toxins have been reported to have antitumoral activity [24].

Although some of these toxins hold promise for developing new cancer pharmaceuticals, several issues must be addressed. The primary concern with using peptides or proteins as active pharmaceutical ingredients (APIs) is their potential for immunogenicity, as well as their impact on bioavailability and pharmacokinetics [25]. One strategy to overcome the aforementioned problems is to encapsulate the molecules into drug delivery systems (DDS), such as polymeric micro- and nanoparticles (NPs) [26]. Polymeric NPs are attractive due to their laudable drug-association properties, relatively simple methods of preparation, controllable size distribution, long-term stability, and suitable surface functionalization. These advanced nanomaterials can protect the active agents from degradation. Moreover, the stimuli-responsive matrix can enhance the controlled-release properties of the drugs, such as specificity, biodistribution, and internalization [27].

In previous research conducted by our group, a simplistic method was developed for the facile and straightforward manufacturing of Cs-*g*-PVCL-based nanoparticles (Cs-*g*-PVCL NPs) [12]. The obtained nanoparticles presented colloidal stability, temperature-controlled drug release (LCST = 39 °C), and the drug-loaded nanoparticles exhibited dose-dependent cytotoxicity (cells can tolerate concentrations as high as 10 mg/mL).

Several studies have demonstrated the potential of using chitosan-based nanoparticles by the ionotropic gelation method for encapsulating venom from various species, including the *Naja naja oxiana* snake [28], the *Crotalus molossus molossus* snake [20], the bee [29], *Vipera albicornuta* [30], and the scorpion [31]. However, despite the importance that stimulus-responsive nanoformulations have gained in recent years, this is the first time that the use of a thermosensitive chitosan derivative has been reported for the entrapment of *C. atrox* venom.

**Scheme 1:**
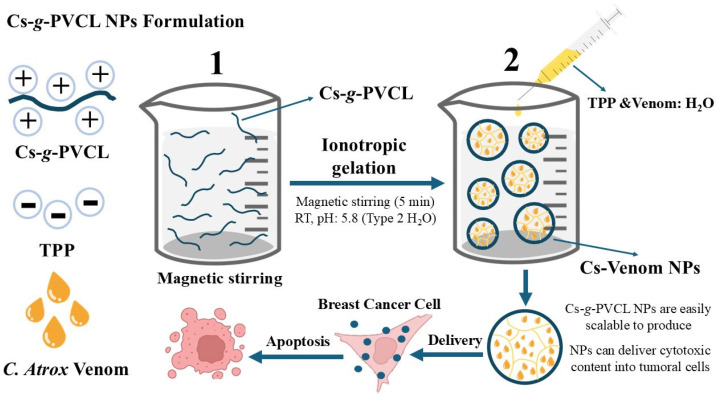
Experimental strategy for formulation of Cs-*g*-PVCL NPs containing *C. atrox* venom by ionotropic gelation. **Step 1**: Dissolution of the copolymer; **Step 2**: Formation of the nanoparticles with TPP containing snake venom.

Thus, this work aimed to prepare biocompatible chitosan-*graft*-poly(*N*-vinyl caprolactam) nanoparticles containing *C. atrox* venom by ionotropic gelation (Scheme 1), and to evaluate this advanced material against two breast carcinoma cell lines as study models for potential drug delivery systems of bioactive components. The influence of the preparation conditions on their physicochemical and biological in vitro properties was also evaluated.

## 2. Materials and Methods

### 2.1. Materials

N-Vinylcaprolactam (98%), 4-(4,6-dimethoxy-1,3,5-triazine-2-yl)-4-methyl morpholinium chloride (DMTMM, 96%), sodium tripolyphosphate (TPP, ≥98%), sodium chloride (≥99%), and glycerol (≥99%) were acquired from Sigma-Aldrich. Isopropanol (98%, Scharlau, Barcelona, Spain), fluorescein isothiocyanate (FITC), dimethylsulfoxide (DMSO, >99.9%, FAGA Lab, Mocorito, Sinaloa, Mexico) fetal bovine serum (FBS; Gibco, Life Technologies Corporation, Grand Island, NY, USA), Fluo 4 AM (Invitrogen, Thermofisher, Waltham, MA, USA), penicillin-streptomycin (Sigma-Aldrich, St. Louis, MO, USA), physiological saline solution (0.9% NaCl, PISA Laboratories, Jalisco, México), potassium chloride (>99%, Meyer, IL, USA), RPMI media (Sigma, St. Louis, MO, USA), sodium pyruvate solution (100 mM, Sigma-Aldrich, St. Louis, MO, USA), thiazolyl blue tetrazolium bromide (MTT, ≥97.5%, Sigma-Aldrich, St. Louis, MO, USA), trypsin (<98%, Sigma, St. Louis, MO, USA) were used as received. 4,4′-Azobis-4-cyanovaleric acid (98%, Sigma-Aldrich, St. Louis, MO, USA) was purified by recrystallization with ethanol. T-47D and MDA-MB-231 cells were obtained from the ATCC (American Type Culture Collection, Manassas, VA, USA).

### 2.2. Ethics Statement

The Research Ethics Committee of the University of Sonora reviewed and approved this research (folio number CEI-UNISON 21/2019). Experiments comply with the principles expressed in the Declaration of Helsinki. Blood was drawn from participants who signed an informed consent form and agreed to have their blood used in hemolysis assays. This study used cells of human ductal carcinoma (T47D) and human adenocarcinoma (MDA-MB-231) as cancer cell lines. Cell lines were purchased from the American Type Cell Culture (ATCC, Manassas, VA, USA). The Itinerant Wildlife Museum (MIVIA) extracted the venom in accordance with the guidelines of the American Society of Ichthyologists and Herpetologists for the use of live amphibians and reptiles. The Secretary of Agriculture, Livestock, Hydraulic Resources, Fishing, and Aquaculture from Mexico (SAGARHPA) issued permits for captive animals and venom extraction for scientific purposes, permits 12/09 00462/15 and DGFF/12/09-1106/18.

### 2.3. Crotalus atrox Venom Extraction and Characterization

The snake used was an adult rattlesnake from the scientific collection of the MIVIA in Hermosillo, Sonora, Mexico. Venom was extracted manually by allowing the snake to bite a sterile 100 mL plastic container covered with parafilm. Afterward, the extracted product was freeze-dried and stored at −20 °C. Before use, venom was resuspended in water and filtered through a 0.2 μm PVDF membrane syringe filter.

Peptide mixtures from venom were analyzed by gel electrophoresis using reducing and denaturing conditions (SDS-PAGE) in a 15% polyacrylamide gel, according to Laemmli [32]. PAGE analysis was performed using 15 µg of venom protein and subsequently stained with Coomassie. The molecular weight of the proteins was estimated by comparing them to an ample range of molecular weight markers (Bio-Rad, Hercules, CA, USA).

### 2.4. Synthesis of Chitosan-graft-poly(N-vinylcaprolactam) Copolymer

The Cs-*g*-PVCL water-soluble copolymer was prepared using the grafting method as previously described [6]. Briefly, PVCL-COOH homopolymer (${\bar{X}}_{n}=190$, polydispersity index = 1.8) [6] was added to the chitosan solution (110 mM) until a homogeneous mixture was obtained. For this purpose, a 2:1 PVCL/Cs feed mass ratio was employed. DMTMM (10-fold molar stoichiometric excess) was used as an activated agent of carboxylic groups in the graft polymerization. The reaction was performed with continual stirring at room temperature for 72 h. Afterward, the mixture was precipitated with an excess of acetone and was purified by Soxhlet extraction with acetone for 48 h. The pure product was dissolved in water and freeze-dried.

### 2.5. Preparation of Nanoparticles

The experimental protocol for preparing Cs-*g*-PVCL NPs by ionotropic gelation was conducted according to a previously established procedure with slight modifications [12,33]. Cs-*g*-PVCL polymer was dissolved (2 mg/mL) in water or aqueous NaCl solutions under continual stirring. Then, a solution of sodium tripolyphosphate (TPP, 4 mg/mL) in the same solvent was used as an ionic crosslinking agent. For the incorporation of venom in the Cs-*g*-PVCL nanoparticles (Venom-loaded NPs), the peptide mixture (5 mg/mL) was previously dissolved in the TPP solution for 2 h. Nanoparticles were formed spontaneously by the dropwise addition of the crosslinker-containing solution (1 mL) into the Cs-*g*-PVCL solution (10 mL) under moderate magnetic stirring at room temperature for 15 min. The Cs-Venom NPs were separated by centrifugation (13,000 rpm, 10 °C, 30 min) using a bed of glycerol (20 μL) previously deposited at the bottom of the vial. The supernatant was carefully removed, and the Cs-Venom NPs were resuspended in water for storage at 4 °C. The recovery yield of the nanoparticles was estimated by comparing the dry weight and the theoretical weight of the Cs-Venom NPs.

### 2.6. Venom-Nanoparticle Entrapment Capacity

The encapsulation efficiency (EE%) of the process was estimated by calculating the difference between the total amount of venom added and the amount of free venom remaining in the supernatant after centrifugation. The concentration of free venom was determined by measuring the absorbance of the supernatant at 280 nm using a UV-Vis spectrophotometer (Thermo Scientific Multiskan GO, Vantaa, Finland). The data were analyzed using SkanIt Software for Microplate Readers (Thermo Scientific, version 7.1). A standard calibration curve for *Crotalus atrox* venom was constructed in the range of 0.02 to 5 mg/mL. Equations (1) and (2) represent the EE% and the loading capacity of nanoparticles (LC%), respectively.

(1) $$EE\%=(\frac{total\;venom-free\;venom}{total\;venom})\times 100$$

(2) $$LC\%=(\frac{total\;venom-free\;venom}{nanoparticle\;weight})\times 100$$

### 2.7. Physicochemical Characterization

Fourier-transform infrared spectroscopy (FTIR) was employed to analyze the chemical composition of nanoparticles and pristine materials. The FTIR spectra of the samples were recorded on a Thermo Scientific Nicolet iS-50 spectrometer (Madison, WI, USA) using the attenuated total reflection (ATR) mode, with 64 scans accumulated at a resolution of 4 cm^−1^.

The average hydrodynamic diameter (D_*H*_), polydispersity index (PDI), and ζ potential of the nanoparticles were characterized by Dynamic light scattering (DLS) on a Möbiusζ equipment (Wyatt Technology, Santa Barbara, CA, USA). The nanoparticles (1 mg/mL) aqueous dispersion was loaded in a quartz cuvette with the electrode assembly at pH 6. The Möbiuζ system uses a 45 mW single-longitudinal-mode laser operating at a wavelength of 532 nm. The measurements were taken at a scattering angle of θ = 163.5°. Before each measurement, the sample temperature was equilibrated to 25 °C. All experiments were carried out in triplicate. Data collection and analysis were performed using DYNAMICS 7.8.0.26 software (Wyatt Technology).

The surface topography of the samples was analyzed in non-contact mode using an atomic force microscope (AFM, Alpha 300RA, WiTec, Ulm, Germany) with nanosensors featuring a spring constant of 42 N/m and a resonant frequency of 285 kHz. An area of 10 × 10 µm was examined. The 2D and 3D images, along with their profiles, were obtained using the WITec project Five 5.1 software (WiTec, Ulm, Germany). Samples were prepared as described previously with 0.4% NaCl. The excess reagents were removed using a dialysis membrane against water for 12 h. Afterward, samples were sonicated for 10 min. Samples were prepared by placing 10 µL of an aqueous dispersion (20 µg/mL) on a microscopic glass slide and allowing it to dry at room temperature before measuring.

Two-dimensional sodium dodecyl sulfate-polyacrylamide gel electrophoresis (2D SDS-PAGE) was performed using a 15% polyacrylamide gel under reducing and denaturing conditions (with SDS and dithiothreitol). Both free venom and venom-loaded NPs were analyzed to determine the molecular masses of the venom proteins and to compare the protein profiles of free and encapsulated venom. Briefly, protein samples (15 µg or 2 µg) from the following conditions were analyzed: crude venom (before and after filtration) and venom extracted from nanoparticles using ultrasonication. Gels were stained with Coomassie Brilliant Blue G-250 (for 10 µg of protein) or silver stain (for 2 µg of protein). Molecular weights were estimated by comparison with a broad-range protein marker (6.5–200 kDa). Protein concentrations in the samples were quantified using the Bradford assay.

### 2.8. Biological Tests

For all biological assays, venom-loaded NPs concentrations were selected based on previous studies involving both plain NPs and NPs encapsulating snake venom. The experiments were carried out based on the concentration of venom encapsulated in the NP. For instance, the highest NP concentration tested (1 mg/mL) contained an equivalent quantity of venom as the highest concentration of free venom used (250 µg/mL). Biocompatibility of the nanoparticles was evaluated using in vitro assays for hemagglutination, hemolysis, and cell viability.

#### 2.8.1. Hemagglutination Assay

To evaluate the biocompatibility of the venom and NPs, a hemagglutination assay was performed as described by Guimaraes-Gomesa et al. [34] with slight modifications. Fresh human blood (blood type O+, drawn from a volunteer) with anticoagulants (ethylenediaminetetraacetic acid, EDTA) was centrifuged, and only the blood cells, without plasma, were used. Red blood cells (RBC) were washed three times with phosphate-buffered saline solution (PBS) by removing the supernatant and resuspending the cells in PBS. A suspension with 2 vol% washed RBC and 0.5 mg/mL Trypsin in PBS was prepared and incubated for 40 min at 37 °C by mixing every 10 min. The sample was rewashed by centrifugation, first for 5 min at 3000 rpm, then three times for 5 min at 2000 rpm, and resuspended in the same volume of PBS. A two-fold dilution series was carried out with venom in concentrations of 250–0.5 µg/mL in duplicate, unloaded NPs, and venom NPs in concentrations of 1000–2 µg/mL in triplicate; a negative control was PBS. Samples were mixed with an equal volume of RBC-trypsin solution in round-bottomed microtiter plates and incubated for 40 min at room temperature. Hemagglutination as the endpoint was identified, and the minimum concentration causing hemagglutination was determined.

#### 2.8.2. Hemolytic Activity

Fresh human blood samples (blood type O+) were collected in EDTA-containing tubes as anticoagulants prior to the assay. Cs-*g*-PVCL NPs and venom-loaded NPs were separated by centrifugation at 13,000 rpm for 30 min at 10 °C, then resuspended in PBS. Crude venom was also dissolved in PBS. Subsequently, two-fold serial dilutions of *C. atrox* venom, Cs-*g*-PVCL NPs, and venom-loaded NPs were prepared in PBS, with concentration ranges of 250–2 µg/mL. Water was used as the positive control (complete hemolysis), and PBS served as the negative control. Blood was added to each tube at a final concentration of 3% *v/v*. The mixtures were gently hand-mixed and centrifuged at 6000 rpm for 1 min. After, the samples were gently remixed and incubated at 37 °C for 24 h. Following incubation, the samples were gently mixed and then centrifuged for 1 min. Hemolysis was first assessed by visual inspection of color change in the supernatant. Quantitative analysis of hemolysis was performed by measuring the absorbance of the supernatant at 540 nm using a UV-Vis spectrophotometer [35]. The percentage of hemolysis was calculated by setting the absorbance of the positive control as 100%.

#### 2.8.3. Cytotoxicity Assay in Breast Cancer Cells

T47D- and MDA-MB-231 cells were grown in RPMI media supplemented with 5% Fetal Bovine Serum (FBS), 1% Penicillin-Streptomycin, and 1% Sodium Pyruvate. Cells were cultured in a humidified atmosphere at 37 °C in the presence of 5% carbon dioxide (CO_2_). The cytotoxicity of unloaded and venom-loaded NPs was determined in triplicate by the 3-(4,5-dimethyl-2-thiazolyl)-2,5-diphenyl-2H-tetrazolium bromide (MTT) assay. The cell lines were cultivated for 24 h in a 96-well plate (Costar, type 3596, Nashville, TN, USA) with an initial cell density of 105 cells mL-1 before treatment. The concentrations used were 250–0.5 µg/mL for the venom and 1000–1.95 µg/mL for both nanoparticle samples (blank NPs and venom-loaded NPs), prepared as two-fold serial dilutions in saline solution. Treatment incubation was carried out using media and the dissolved stimuli in a 1:2 dilution with a final concentration of 5% FBS. Afterward, cells were incubated for 24, 48, or 72 h. Treatment doses of NPs were chosen based on earlier studies with venom, and venom NPs and venom concentrations were selected by calculating the concentrations of entrapped venom in the NPs. The saline solution was used as a negative control. After incubation with MTT, dimethyl sulfoxide was used to dissolve the crystals. The cell morphology was observed using an inverted microscope (Nikon, Eclipse Ti-U, Tokyo, Japan), and cells were compared to the control groups. Additionally, a comparison of the morphological changes and cell viability was conducted. The optical density was measured using a Multiskan GO reader (Thermo Fisher, Vantaa, Finland), and absorbance was measured at 570 nm. Cell viability was calculated using the following equation, with control values set at 100% viability. Cell viability and cytotoxicity were estimated using Equation (3).

(3) $$Cell\;viability\left(\%\right)=\frac{{Absorbance}_{sample}}{{Absorbance}_{control}}\times 100$$

### 2.9. Intracellular Calcium in T47D Cell Cultures

Cultures of T47D cells were prepared as follows: first, T47D cells were seeded on a sterile glass coverslip (40,000 cells/mL) and placed inside a sterile 35 mm Petri Dish with 3 mL RPMI media supplemented with 5% FBS, penicillin-streptomycin (1%), and sodium pyruvate (1%). After 24 h, a piece of the glass coverslip containing attached-T47D cells was rinsed with saline solution (SS, composed in mM: 135 NaCl, 5.4 KCl, 5 HEPES, 1.8 CaCl_2_, 1 MgCl_2_, 10 glucose, pH 7.2) and then incubated with 1 μM Fluo 4 AM diluted in SS for 20 min in a dark room to address if the snake venom would produce intracellular Ca^2+^ ([Ca^2+^] i) responses in T47D cell cultures. After that, an excess of Fluo 4 was rinsed with SS, and T47D cells were placed on a custom-made perfusion system positioned on the platina of an inverted confocal microscope (Nikon Eclipse C2+). The cells were continuously perfused with SS at room temperature to record [Ca^2+^] i responses. T47D cells were observed through a plan-apochromat 10×/0.8 NA objective.

[Ca^2+^] i responses were recorded by scanning frames with 1024 × 1024-pixel resolution every 1 s for 12 min to investigate the effect generated on T47D cells by the snake venom. The excitation wavelength was 488 nm (Argon laser), and the emission was collected at 510 nm with a dichroic mirror. Laser intensity was kept at <10% to protect the samples. [Ca^2+^] i responses are depicted as fluorescence intensity ratio (arbitrary units, AU). The fluorescence data and image analysis were acquired on the NIS Elements Viewer software Nikon Instruments Inc (version V. 5. 21.00). Data were processed and illustrated with ImageJ-FIJI (Version 1.54p) and Origin Pro 8.2 software. Results are presented as mean ± standard error of at least 20 T47D cell recordings, whether as a confluent or isolated group of cells.

For this type of experiments, the snake venom dissolved at 60 μg/mL (determined by IC_50_) in SS was applied to T47D cells as follows: first, T47D cells loaded with Fluo 4 were recorded for 3 min in SS, then 20 mM KCl was perfused for 20 s, and after that, the cells washed out with SS for 3 min, then 20 µL of venom was applied directly to the perfusion system and washed out for 6 min. To test the effect of NPs loaded with snake venom, T47D cells placed in the perfusion chamber were incubated with the NPs for 45 min, and every 15 s, a frame of 1024 × 1024 pixels resolution was recorded.

### 2.10. Statistical Analysis

In this work, all samples were analyzed in triplicate, and the data are presented as the mean ± SD. The significance of the differences among the groups was analyzed using one-way ANOVA with Dunnett’s multiple comparisons test, as performed with GraphPad Prism 7.0 software (San Diego, CA, USA). GraphPad Prism was used to calculate the 50% inhibition of cell proliferation (IC50) using nonlinear regression with a sigmoidal four-parameter logistic model for the cytotoxicity assay.

## 3. Results and Discussion

### 3.1. Characterization of C. atrox Venom

Comprehensive characterization of the venom is essential for advancing its potential pharmaceutical applications. Therefore, our study began with a detailed analysis of the venom through multiple complementary approaches. The venom protein composition (%) was first determined using a UV-VIS spectrophotometer by comparing the absorbance values with a previously established protein calibration curve. We estimated that 82 ± 2.8% of the dried venom was protein. Snake venom milking was performed traditionally; therefore, we filtered it through a 0.2 µm filter to eliminate undesirable residues. DLS characterized the samples to determine the distribution and size differences between the crude and filtered venom samples, as shown in Table 1.

The filtered venom significantly reduced its hydrodynamic diameter (D_*H*_), decreasing from 1618 nm to 175 nm. This can be easily explained when considering that venom extraction may be followed by organic material, such as cells, bacteria, and other components that the DLS analysis may classify as large particles. The polydispersity index (PDI) also decreased its value from 0.60 to 0.22, indicating a more homogeneous sample after filtration. On the other hand, the ζ-potential did not have any significant changes; this could be explained by considering that snake venoms are complex mixtures of several peptides and proteins, each with different ζ-potentials.

**Figure 1:**
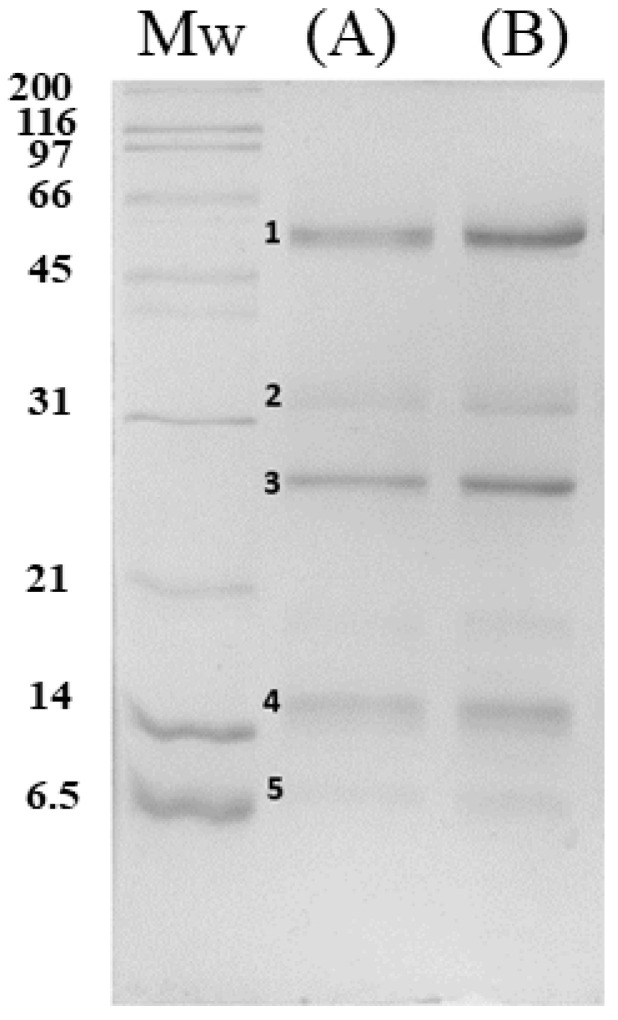
15% SDS-PAGE gel electrophoresis of the venom of *C. atrox*. Molecular weight (Mw) marker, (**A**) unfiltered venom, (**B**) filtered venom. 15 µg of protein were loaded for evaluation.

We evaluated the filtered and unfiltered venom using SDS-PAGE gel electrophoresis for further characterization and visualization of protein bands. The experiment was conducted under reducing and denaturing conditions to observe the protein band pattern (Figure 1). Five protein bands were observed with a molecular weight of around (1) 66–45 kDa, (2) 34–32 kDa, (3) 30–25 kDa, (4) 17–15 kDa, and a slight band at (5) 8–7 kDa. Bands (1) and (3) were the most abundant. The results indicate that no apparent protein bands were lost, showing that filtering the venom of *C. atrox* does not change the venom composition by removing specific proteins.

Calvete et al. [22] previously reported that the venom proteome of *C. atrox* is a mixture of several protein families. The significant components are snake venom metalloproteases (SVMPs) and snake venom serine proteases (SVSPs), which comprise ~70% of the venom, consisting of 24 detected proteins. Approximately 26% of the venom composition belonged to what they referred to as medium-abundance families, such as A2 phospholipases (PLA2s), disintegrins (DIS), L-amino acid oxidases (LAAOs), and cysteine-rich secretory proteins (CRiSPs). It is important to note that this analysis does not confirm which protein bands observed herein belong to a specific toxin family but confirms that no protein bands were lost during filtration.

### 3.2. Physicochemical Characterization of Nanoparticles

The obtention of Cs-*g*-PVCL NPs by ionotropic gelation using TPP as a cross-linking agent has been previously studied [12,33]. It has been found that the size of the particles is highly dependent on the chitosan to TPP molar ratio, initial chitosan concentration, and the degree of acetylation of the chitosan. Interestingly, Sreekumar et al. [36] reported that the particle formation and the hydrodynamic diameter of the chitosan-based NPs are strongly dependent on the ionic strength in the medium [36]. Therefore, we investigated the effect of NaCl concentration on the formation of Cs-*g*-PVCL NPs (Table 2).

The presence of NaCl in the nanoformulations strongly influences the hydrodynamic size of the particles. It is possible to observe that at a low concentration of salt (0.4%), the average hydrodynamic diameter of particles decreased radically, and their size distribution became more uniform. In contrast, both parameters tended to increase with higher concentrations. These results are in agreement with reports found in the literature for chitosan-based nanoparticles obtained by this method [1,36]. The presence of NaCl favors a reduction in the stiffness of the macromolecular chains and the charge repulsion between chitosan chains, leading to the formation of more compact particles [36]. This effect is more significant in the venom-loaded NPs due to the charged substance containing a high number of proteins.

**Figure 2:**
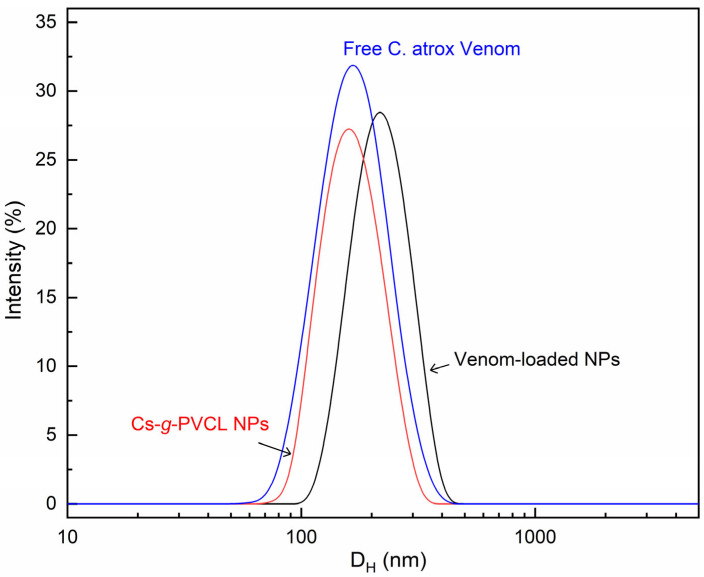
Graphical hydrodynamic size distribution for filtered *C. Atrox* venom, Cs-*g*-PVCL NPs, and Venom-loaded NP. All samples were evaluated in a 0.4% NaCl saline solution.

With increased salt solution up to 0.8% NaCl (147 mM), the venom-loaded NPs slightly increased in size and PDI. It can also be observed that the ζ-potential decreases at higher amounts of NaCl; this effect was also observed in Cs-*g*-PVCL NPs. There were no significant differences in the EE% between the venom-loaded NPs with and without NaCl in their formulation. Therefore, from this point onward, all the results presented are from the venom-loaded NPs with 0.4% NaCl. Figure 2 illustrates the graphical size distribution for free venom, Cs-*g*-PVCL NPs, and Venom-loaded NP.

Particle sizes between 50 and 300 nm in diameter have been shown to have longer circulation times and less accumulation in the spleen and liver compared to larger particles [15,37]. For example, it has been reported that small-sized NPs allow them to distribute more effectively in the body and penetrate directly through leaky vessels into tumor tissue [15]. Additionally, the bigger a particle is, the easier it is for the immune system to recognize it, hence the importance of controlling the NP size.

Following the importance of NP DH, the ζ-potential, or surface charge, should not be overlooked. For general purposes, it has been recognized that with charged particles, ζ-potential values of >±15 mV are generally considered stable particles. Additionally, positively charged particles have been reported to exhibit increased cellular uptake, which allows the drug carrier to enter the cell more efficiently and develop a more significant pharmaceutical effect [15].

Other researchers have prepared venom-loaded NPs with a size between 120 and 200 nm. Mohammadpour Dounighi et al. [28] reported the preparation of chitosan-based NPs with Naja naja oxiana snake venom sizes ranging from 120 to 150 nm and an EE% of 89.9%. Similarly, Mirzaei et al. [38] obtained nanoparticles with a length of 182 nm and a loading efficiency of 94%. Soares et al. [39] also prepared particles in a size range of 160–200 nm using different venoms from Bothrops species; depending on the venom concentration used, they reported an EE% of 68–97%.

**Figure 3:**
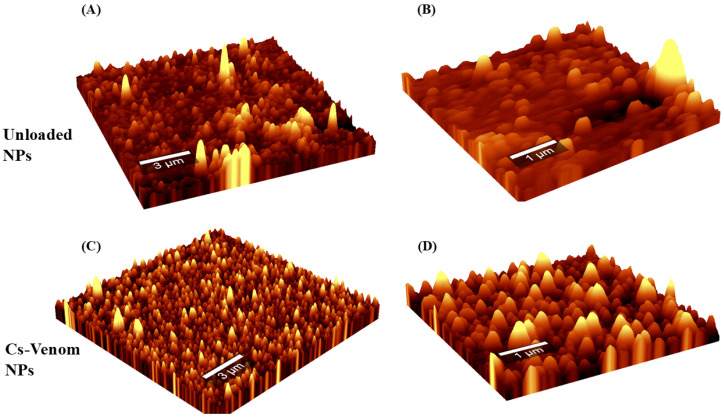
AFM images in non-contact mode of Cs NPs in (**A**) 10 × 10 µm and (**B**) 3 × 3 µm, and Cs-Venom NPs in (**C**) 10 × 10 µm and (**D**) 3 × 3 µm.

To study the topography and surface of the samples, Cs-*g*-PVCL NPs (Figure 3A,B) and Venom-loaded NPs (Figure 3C,D) were analyzed by AFM in non-contact mode. Both systems have a smooth surface with a spherical-like shape. It is possible to perceive that the diameter of the blank NP was approximately 185 nm, while the venom-loaded NPs showed sizes in the order of 230 nm. Our group previously reported that venom-loaded NPs, with surfaces and shapes similar to each other, although the sizes were significantly larger [20]. It is essential to consider that the AFM non-contact mode may enhance the overall size of the reconstructed image. Likewise, regularity is observed in the sizes and shapes of the particles obtained, which is typical of systems formed through ionotropic gelation.

**Figure 4:**
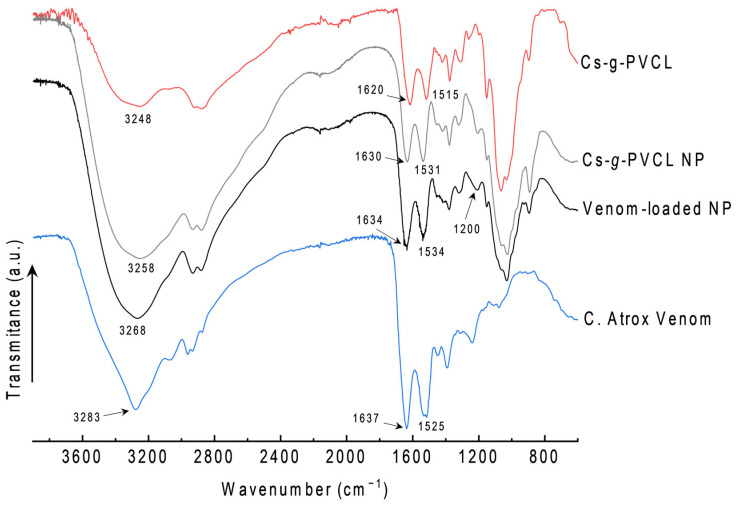
FTIR spectra in the region 4000–600 cm^−1^ for Cs-*g*-PVCL, Cs-*g*-PVCL NP, Venom-loaded NP, and Venom.

The chemical structural characterization of nanoparticles was performed using FTIR spectroscopy. Figure 4 displays the spectrum of venom, chitosan-*g*-PVCL, Cs-*g*-PVCL NP, and venom-loaded NP. Cs-*g*-PVCL exhibits the bands centered at 1620 cm^−1^ (amide I), 1515 cm^−1^ (amide II), 1415 cm^−1^ (CH_2_ bending), 1376 cm^−1^ (CH_3_ symmetrical deformation vibration), 1308 cm^−1^ (amide III), 1151 cm^−1^ (C-O-C asymmetric stretching vibration), and 1066 and 1031 cm^−1^ (vibration of the pyranose structure), 896 cm^−1^ (D-glucopyranose ring stretching) [40].

The spectrum of the Cs-*g*-PVCL NPs shows a similar pattern to that of the pristine copolymer. A shift in the Amide II band towards 1531 cm^−1^ is observed, which can be attributed to the interaction of the amino groups with the phosphate groups of TPP. The band displayed at 1206 cm^−1^ is attributed to the P=O stretching of TPP in the NPs [12,31]. On the other hand, *C. atrox* venom showed bands at 3283 cm^−1^, which is due to the N-H stretching vibration associated with intramolecular hydrogen bonding. Subsequently, the characteristic bands of the polypeptides are found at 1637 cm^−1^ (amide I), 1525 cm^−1^ (amide II), 1388 cm^−1^ (CH_3_ symmetrical bending), 1240 cm^−1^ (primary aliphatic amines), and 1077 cm^−1^ (CO–O=C symmetrical stretching). The position of the amide I and amide II bands suggests an extended chain form (β-form) in the secondary structure of the protein [40]. Regarding the venom-loaded nanoparticles, the spectra exhibited a very similar pattern to that of the unloaded nanoparticles. These results align with reports for similar systems [28,41]. In another sense, when comparing the free venom and its loaded form in nanoparticles, some significant changes can be observed. In the 3200–3300 cm^−1^ region (N–H stretching vibration), the band observed in the loaded NPs is very wide, which could overlap the band corresponding to the free polypeptide. Likewise, in the case of the band found at 1525 cm^−1^ (amide II) for the free venom, a slight shift to the left is observed (to 1534 cm^−1^), which could be related to interchain interactions with the polymer matrix molecules [40]. This behavior suggests that the polymer matrix remains mainly on the surface of the nanoparticles, while the majority of the venom molecules are immobilized internally. This observation is feasible, since the ζ-potential of the loaded NPs is not significantly affected by the incorporation of polypeptide chains, maintaining positive values corresponding to the chitosan derivative (see Table 2).

**Figure 5:**
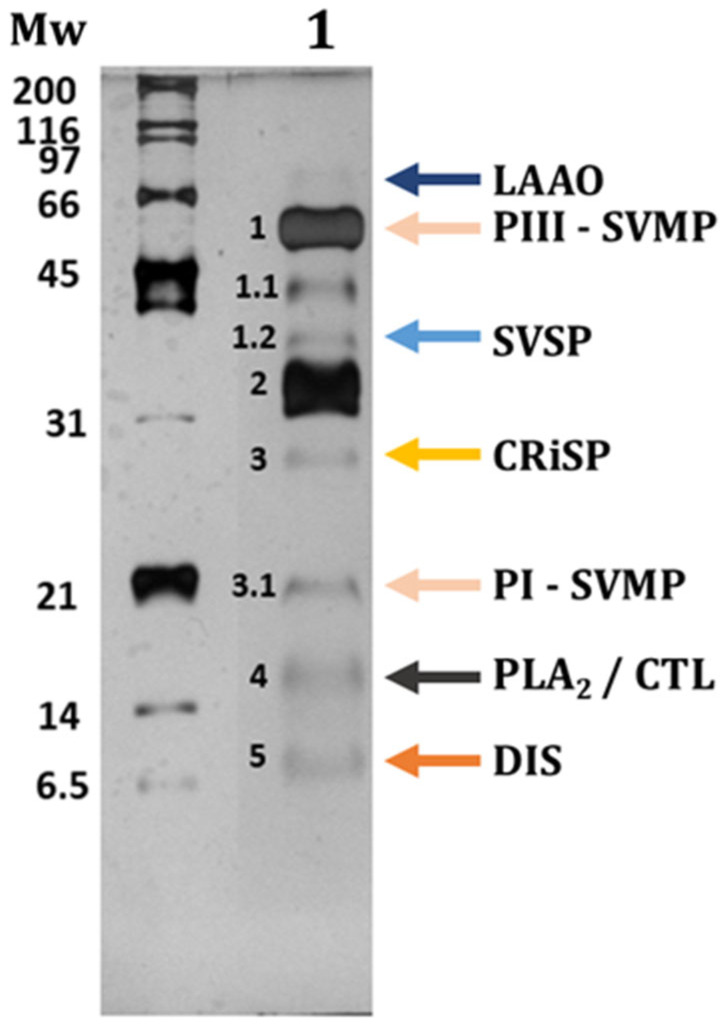
Graphic representation of the diversity of toxins from *C. atrox* venom. 15% SDS-PAGE gel of venom (2 µg) with silver staining. Columns in the SDS-PAGE gel are arranged as molecular weight (MW) markers, and 1. released venom from the Cs-Venom NPs.

To confirm the entrapment of the previously observed protein bands from the SDS-PAGE gel electrophoresis of the filtered venom, we analyzed the venom released by ultrasonication from the Venom-loaded NPs (Figure 5). Three additional bands (1.1, 1.2, and 3.1) were observed in this gel compared to those in the venom alone (see Figure 1). The reason could be that the silver staining is more sensitive than the Coomassie blue G250, hence detecting low quantities of protein bands. This analysis confirmed that Venom-loaded NPs can encapsulate different proteins with a wide range of molecular weights (MW). In this sense, Rex and Mackessy [23] characterized several venoms of adult *C. atrox* using different methods, including SDS-PAGE gels with silver staining. They reported practically the same bands that we observed, with slight differences. Considering their results, we infer that the protein band, barely visible at ~66 kDa, is a LAAO. Band 1 corresponds to a PIII-SVMP, and bands designated as 1.1, 1.2, and 2 may correspond to SVSP. Band 3 corresponds to a CRiSP, and finally, bands 3.1, 4, and 5 to a PI-SVMP, a PLA2/CTL, and DIS, respectively.

### 3.3. Hemagglutination and Hemolytic Activity Assays

**Figure 6:**
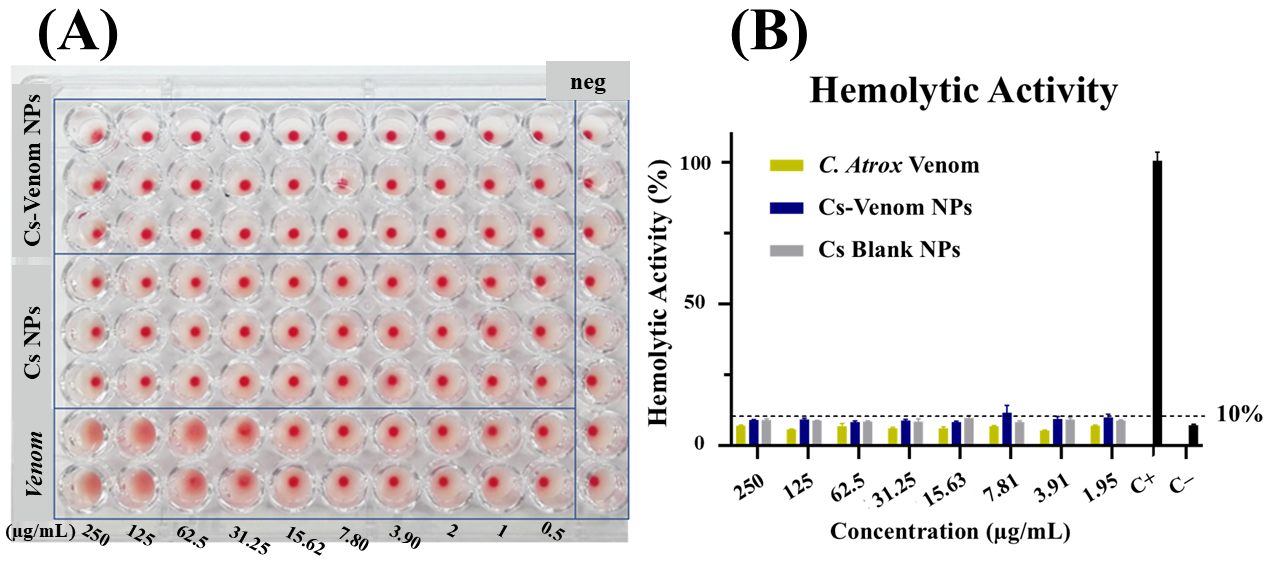
Biocompatibility assays in RBC for *C. atrox* venom, Cs-*g*-PVCL NPs, and Venom-loaded NPs: (**A**) Hemagglutination assay. Negative control (neg) (PBS) on the right; and (**B**) Hemolytic activity. Data are means ± standard deviation.

Biocompatibility is a fundamental property of biomaterials that enables understanding of their biological response when in contact with living tissues. To this end, nanoparticles were evaluated using in vitro assays for hemagglutination, hemolysis, and cell viability. Figure 6 shows the results of the hemagglutination activity of *C. atrox* venom, Cs-*g*-PVCL NPs, and Venom-loaded NPs. Snake venom agglutinated RBC at concentrations greater than 31.25 µg/mL; in contrast, nanoparticle systems did not exhibit any hemagglutination or hemolysis at the same concentrations.

It has been reported that *C. atrox* venom contains C-type lectins (CTLs) (abundance of around ~1.7%) with high hemagglutination potential [22]. From Figure 6A, we infer that the band observed at ~4 kDa could be a CTL. Hirabayashi et al. [42] evaluated *C. atrox* venom CTLs and reported that only 0.6 ng/mL of purified snake lectin was needed to present hemagglutination activity in erythrocytes. Additionally, several researchers have reported that the minimum concentration of purified CTLs for hemagglutination activity can range from 0.02 to 20 µg/mL [34,43,44,45,46].

The analysis shows no apparent hemagglutination activity from Cs-*g*-PVCL NPs and Venom-loaded NPs. Furthermore, the biocompatibility of both Cs-based derivatives has been previously reported [47,48], which supports our findings.

Continuing with the biocompatibility tests, the hemolytic activity of *C. atrox* venom, Cs-*g*-PVCL NPs, and Venom-loaded NPs was evaluated after treatment with RBC (Figure 6B). It is evident that the value of 10% is never exceeded.

When evaluating materials, a 10% hemolytic activity value is considered a threshold to categorize them as non-hemolytic, while those exceeding 20% are considered potentially hemolytic. As shown in Figure 6, none of the evaluated treatments exceeded the 10% HA threshold.

Lima et al. [49] reported that Cs NPs showed no hemolytic activity. Studies previously conducted by our laboratory evaluated the hemolytic activity of Cs NPs loaded with Crotalus molossus venom [20]. In this report, it was found that a higher concentration range (3000—46 µg/mL) was associated with no hemolytic activity, as reported at concentrations below 200 µg/mL of venom. Furthermore, no hemolytic activity surpassing the 10% threshold was observed with the Venom-loaded Cs NPs.

The qualitative analysis found a slight hemolytic activity in the negative control and the treated RBC. This can be explained by the physiological natural lysis caused by the death of some RBC and by slight hemolysis at blood collection [50].

Higher hemolytic activity could be expected from evaluating *C. atrox* venom, considering the presence of PLA2s and LAAOs; both toxins have been reported to cause hemolysis [24,35,51,52]. Nevertheless, it is essential to consider the relatively small percentage of the toxin mentioned above in these families, in contrast to others, such as the PIII-SVMP and SVSP, as observed in the SDS-PAGE graphical representation of venom (see Figure 5).

### 3.4. MTT Cell Viability Assays

**Figure 7:**
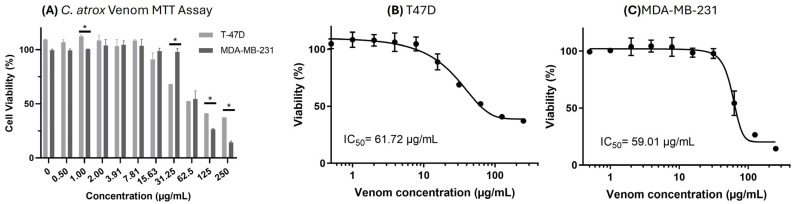
Graphic representation of the (**A**) Cell viability (%) of T47D and MDA-MB-231 cell lines with *C. atrox* venom. Individual graphic representation of the IC50 of (**B**) T47D and (**C**) MDA-MB-231 in a non-linear regression. Data is expressed as means ± standard deviation. Significance is shown as asterisks: * *p* ≤ 0.05.

The cytotoxic activity of *C. atrox* venom, Cs-*g*-PVCL NPs, and Venom-loaded NPs was analyzed in two breast cancer cell lines (T47D and MDA-MB-231). Figure 7 shows the cell viability of the cell lines mentioned in relation to *C. atrox* venom.

To the best of our knowledge, no articles have reported the IC_50_ values of the venom of *C. atrox* in T47D and MDA-MB-231 breast carcinoma cell lines. In Figure 7, it can be observed that the snake venom is cytotoxic at high concentrations (~250 µg/mL), and a 50% cell viability is achieved in the range of 31–125 µg/mL. Additionally, we determined the IC_50_ values for the *C. atrox* venom in these two cell lines by analyzing cell viability using nonlinear regression. We determined the IC_50_ values for T47D and MDA-MB-231 to be 61.72 and 59.01 µg/mL, respectively, with no significant differences. This analysis confirms that rattlesnake venom retains its bioactivity after lyophilization and filtration.

In comparison, Jimenez-Canale et al. [20] found the cytotoxic activity of the northern black-tailed rattlesnake (*C. molossus*) in T47D cells, with an IC_50_ of 15.45 µg/mL. The cytotoxicity of snake venom depends on the snake species itself, as it determines the venom composition and the cell line being evaluated. For example, Bradshaw et al. [53] reported that the venom of *C. cerberus* and *Bothrops alternatus* had IC_50_ values of 17.5 µg/mL and 63.5 µg/mL, respectively, in the human breast adenocarcinoma cell line MCF-7. On the other hand, Yalcin et al. [54] also evaluated the venom of the *Ottoman viper*, *Montivipera xanthina*, in MCF-7 cells, reporting an IC_50_ of 4.2 µg/mL, which is significantly lower than the values reported for other snake species in the same cell line.

Cytotoxicity studies of Cs-*g*-PVCL NPs have been previously reported by our research group [12]. Additionally, Cs NPs have been reported to interact with the cell membranes of both normal and cancer cells through electrostatic interactions, thereby inducing their internalization within lysosomes [55,56].

Huang et al. [56] reported that the internalization rate of Cs NPs may be increased depending on the degree of deacetylation, the molecular weight of the Cs used, and the ζ-potential. Highly positive Cs micro- and nanoparticles with a ζ-potential ≥ may increase the internalization rate. Additionally, spherical and smooth-surfaced NPs with a DH < 500 nm have been reported to help exploit the enhanced permeability and retention (EPR) effect in solid tumors (internalizing them at higher rates) [57]. Therefore, we evaluated the cytotoxicity of the Venom-loaded NPs to analyze their potential as a bioactive agent for drug delivery.

**Figure 8:**
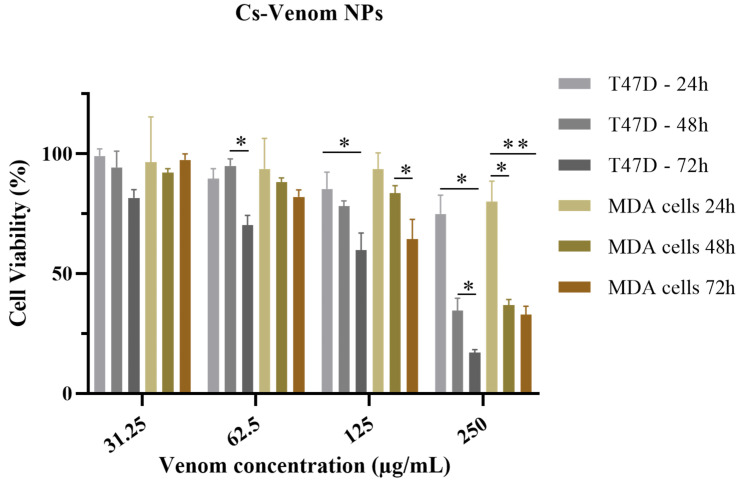
Evaluation of the cytotoxic activity of Venom-loaded NPs in T47D and MDA-MB-231 at 24, 48, and 72 h. Data are expressed as means ± standard deviation. Significance is shown as asterisks: * *p* ≤ 0.05; ** *p* ≤ 0.01.

Figure 8 shows the results of the cytotoxicity assay for Venom-loaded NPs in both breast carcinoma cell lines up to 72 h. The lowest cell viability is reported in both cell lines at 250 µg/mL and 72 h. Furthermore, although *C. atrox* venom IC_50_ (61.72 and 59.01 µg/mL for T47D and MDA-MB-231 cells, respectively) was evaluated, cell viability was not reduced to ~50% until the next higher concentration (125 µg/mL).

Cs-based NPs can be internalized by electrostatic interactions between the overall negative potential of the cell membrane and their highly positive surface [58]. Notably, some NPs may accumulate extracellularly and release the venom over time at the membrane, rather than intracellularly [59,60]. Following that, it is possible to suggest that the cytotoxicity observed in Figure 8 from the Cs-Venom NPs is due to the toxins released from the polymer matrix.

The observed cytotoxic effects of *C. atrox* venom and Venom-loaded NPs can be attributed to the activity of different toxin families that comprise them. For example, SVMPs can interfere with cell adhesion and induce morphological changes in cells; DISs inhibit cell–cell adhesion and migration; LAAOs initiate apoptosis through the generation of free radicals and membrane permeabilization, and PLA2s cause nonspecific membrane damage [24,51,61,62,63,64]. This corroborates the result shown in the SDS-PAGE graphical representation of *C. atrox* venom (see Figure 5), indicating that the polymer-based NPs can encapsulate all the protein bands from the snake venom, specifically PIII-SVMP and SVSP, which are the most abundant proteins.

Further studies are required to confirm the internalization and kinetic release profile of the mentioned NPs. *C. atrox* venom was less cytotoxic than other evaluated snake venoms in different cell lines, as previously mentioned; nevertheless, although its IC_50_ was evaluated at different time rates, a 50% cell viability was not reached, indicating a possible interaction between the venom proteins and the polymer matrix. In that matter, Venkatesan et al. [65] treated *Daboia russelii* venom-loaded CS NPs with the enzyme chitosanase for NP degradation. They reported that using 500 µg/mL and 1000 µg/mL of chitosanase resulted in the release of only 51% and 69% of the encapsulated snake venom, respectively. Their results could help indicate why our study did not observe the same cytotoxicity from *C. atrox* venom and the Cs-Venom NPs at the same concentrations. Considering that our NPs possessed all the qualities for increased internalization, a strong ionic interaction between the encapsulated proteins and the polymer matrix could explain the issue with cell viability.

### 3.5. Tumoral Cells Morphological Changes

The morphological changes caused by *C. atrox* venom and Venom-loaded NPs were also analyzed and shown in Figures S1 and S2 for T47D and MDA-MB-231, respectively. Healthy, untreated T47D and MDB-MB-231 cells were spindle-shaped. When treated with venom-loaded NPs, both cell lines showed morphological changes, especially at the highest concentration of 250 µg/mL.

Morphological changes were observed throughout the MTT assays when the venom and Venom-loaded NPs were applied. The changes became more noticeable over time, as evidenced by the following progression: 24 < 48 < 72. Considering that T47D and MDA-MB-231 are adherent cells, losing their capacity to adhere to a substrate is a common sign of dying cells. In this regard, the results obtained in Figures S1 and S2 exhibit the cellular death mechanism that may be occurring with both cell lines. According to the SDS-PAGE silver-stained gel we reported, SVMP and SVSP are the leading protein bands presented in the venom and encapsulated. In their review, Calderon et al. [24] reported the antitumoral capacity of these toxin families by acting on extracellular matrix components, such as fibrin and collagen, among others.

Tanjoni et al. [66] evaluated an SVMP in endothelial cells, noting that the drastic morphological changes and loss of adhesion could be interpreted as a loss of cell function and the initiation of an apoptotic process. They also reported rearrangements in the actin network, which can lead to cell damage and apoptosis. Other studies have shown that snake venoms, particularly the protein group of metalloproteinases, can be associated with these changes [67,68]. Further studies are needed to determine if any significant morphological changes or loss of cell function are induced in non-transformed cell lines, thereby supporting the safety of using these polymeric NPs as bioactive drug delivery systems.

### 3.6. Intracellular Calcium Concentrations

To study the effects of snake venom on T47D cells, we perfused both the *C. atrox* venom and the Venom-loaded NP into these cells, which were loaded with Fluo 4 AM (an intracellular Ca^2+^ indicator). Both approaches produced sustained intracellular Ca^2+^ responses in T47D cells, suggesting that the snake venom induces intracellular Ca^2+^-associated metabolism that requires further investigation (Figure S3).

In another set of experiments, T47D cells were incubated with Venom-loaded NPs, and the effects were recorded over a 40-min period. Cells showed a similar sustained intracellular Ca^2+^ rise as cells treated only with the snake venom (as in B). Furthermore, the cells exhibited slight morphological changes, including shrinkage and a slightly more spherical shape.

## 4. Conclusions

Drug delivery systems may enhance the therapeutic activity of already-used pharmaceuticals. This work demonstrates the entrapping of *C. atrox* venom into Cs-*g*-PVCL nanoparticles using a facile and straightforward ionotropic gelation process. Venom-loaded NPs had a spherical shape and smooth surface. Adding NaCl to the copolymer reduced the hydrodynamic size of NPs and improved their polydispersity. Hemagglutination and hemolytic experiments suggest that Venom-loaded NPs release the toxins progressively, which can be attributed to the thermosensitive nature of the polymeric matrix. Thus, the biological and physicochemical characteristics offered by *C. atrox* venom entrapment with Cs-*g*-PVCL NP offer a potentially advantageous alternative to chemotherapeutic agents, as they tend to have highly specific targets and few side effects due to off-target cytotoxic effects. Nonetheless, this new advanced material warrants further investigation to assess its broad capabilities as an alternative treatment for various types of cancer.

## Figures and Tables

**Table 1:** Particle size characteristics and ζ-Potential data from crude and filtered venom of *C. atrox*.

Sample	D_*H*_ (nm)	PDI	ζ-Potential (mV)
Crude venom	1618 ± 112	0.60	−4.2 ± 0.4
Filtered venom	175 ± 2.7	0.22	−5.0 ± 0.2

**Table 2:** Characteristics of Cs-*g*-PVCL nanoparticles obtained by ionotropic gelation.

Sample ^a^	D_*H*_ (nm)	PDI	ζ Potential (mV)	EE (%)	LC (%)	Yield (%)
Cs-*g*-PVCL NP	244 ± 1.2	0.28	+ 30.8 ± 0.7	-	-	89.0
Venom-loaded NP	628 ± 2.8	0.90	+ 31.7 ± 0.8	88.0 ± 2.2	22 ± 1.6	86.5
Cs-*g*-PVCL NP—0.4% NaCl	198 ± 0.9	0.19	+ 21.8 ± 2.1	-	-	90.0
Venom-loaded NP—0.4% NaCl	222 ± 1.1	0.24	+ 32.0 ± 1.1	88.6 ± 0.6	22.3 ± 0.8	84.0
Cs-*g*-PVCL NP—0.8% NaCl	262 ± 1.2	0.15	+ 17.5 ± 2.2	-	-	88.2
Venom-loaded NP—0.8% NaCl	283 ± 1.6	0.47	+ 25.4 ± 1.4	85.1 ± 1.5	21.2 ± 0.6	85.3

^a^ All formulations were prepared using a copolymer/TPP mass ratio of 4:1, and the volume ratio of the copolymer/TPP solutions was 10:1.

## Data Availability

Data that support the findings of this study are available within the article and from the corresponding author upon request.

## Supplementary Materials

The following supporting information can be downloaded at: https://www.mdpi.com/article/10.3390/nano15191538/s1, Figure S1: T47D cells were treated with venom NPs with a resolution of 20× in a light microscope after (A) 24 h, (B) 48 h, and (C) 72 h. Concentrations used were (1) 0, (2) 250, (3) 125, (4) 62.5, and (5) 31.25 µg/mL. Morphological changes were indicated with arrows.; Figure S2: MDA-MB-231 cells were treated with venom-loaded NPs with a resolution of 20x in a light microscope after (A) 24 h, (B) 48 h, and (C) 72 h. Concentrations used were (1) 0, (2) 250, (3) 125, (4) 62.5, and (5) 31.25 µg/mL. Morphological changes were indicated with arrows.; Figure S3: T47D cells loaded with 1 M Fluo 4 AM. Intracellular Ca^2+^ imaging of T47D cells superfused with Saline Solution (A, control) and during the snake venom application (venom dissolved in saline solution, (B). As illustrated in B, the intensity of Fluo 4 (arbitrary units) increased in all cells. This intracellular Ca^2+^ rise persisted throughout the entire experiment (up to 12 min). These experiments represent n > 20 T47D cells.

## Abbreviations

The following abbreviations are used in this manuscript:

Cs-*g*-PVCL	chitosan-*graft*-poly(*N*-vinylcaprolactam) copolymer
PVCL	poly(*N*-vinylcaprolactam)
Cs-*g*-PVCL NPs	chitosan-*graft*-poly(*N*-vinylcaprolactam) nanoparticles
Cs	Chitosan
Venom-loaded NPs	chitosan-*graft*-poly(*N*-vinylcaprolactam) nanoparticles loaded with *C. atrox* snake venom
NPs	nanoparticles
LCST	Lower critical solution temperature
*C. atrox*	*Crotalus atrox* snake
D*_H_*	Average hydrodynamic diameter
PDI	Polydispersity index
TPP	Sodium tripolyphosphate
RBC	Red blood cells
