# Supplementary material for: Chitosan-graft-poly(N-vinylcaprolactam) Nanoparticles Containing Crotalus atrox Snake Venom: Biological and Physicochemical Characterization
Source: Nanomaterials (Basel). 2025 Oct 9;15(19):1538. doi: 10.3390/nano15191538 (PMC12525661; doi:10.3390/nano15191538)
Supplement: Supplementary file 1 [file nanomaterials-15-01538-s001.zip › nanomaterials-3889840-supplementary.pdf]

Chitosan-*graft*-poly(*N*-vinylcaprolactam) Nanoparticles containing *Crotalus atrox* Snake venom: Biological and Physicochemical Characterization

Serena S. Rudy [1,‡], Jorge Jimenez-Canale [2,‡], Jose A. Sarabia-Sainz [3,*], Ana M. Guzmán Partida [4], Alexel J. Burgara-Estrella[3], Erika Campa-Silva[3], Aracely Angulo Molina [3,5], Marcelino Montiel-Herrera [6], Nelly Flores-Ramírez [7], Paul Zavala-Rivera [8], and Daniel Fernandez-Quiroz [8,*]

[1] School of Life Sciences, University of Applied Sciences and Arts Northwestern Switzerland, Hofacker Strasse 30, 4132 Muttenz, Switzerland
[2] Department of Research in Polymers and Materials, University of Sonora, Hermosillo 83000, Sonora,
Mexico; jorgejimzc@gmail.com
[3] Department of Research in Physics, University of Sonora, Hermosillo 83000, Sonora, Mexico; alexel.burgara@unison.mx (A.J.B.-E.); erika.silva@unison.mx (E.C.-S.)
[4] Center for Research in Food and Development, Food Science Research Group, Gustavo Enrique Astiazaran Rosas 46, Hermosillo 83304, Sonora, Mexico; gupa@ciad.mx
[5] Department of Chemical-Biological Sciences. University of Sonora, Hermosillo 83000, Sonora, Mexico; aracely.angulo@unison.mx
[6] Department of Medicine and Health Sciences, University of Sonora, Hermosillo 83000, Sonora, Mexico; marcelino.montiel@unison.mx
[7] Department of Wood Engineering and Technology, Universidad Michoacana de San Nicolás de Hidalgo, Morelia 58030, Michoacán, Mexico; nelly.flores@umich.mx
[8] Department of Chemical Engineering and Metallurgy, University of Sonora, Hermosillo 83000, Sonora, Mexico; paul.zavala@unison.mx
* Correspondence: daniel.fernandez@unison.mx (D.F.-Q.); jose.sarabia@unison.mx (J.A.S.-S.).
† In memory of Prof. Martín Rafael Pedroza Montero.
‡ These authors contributed equally to this work.

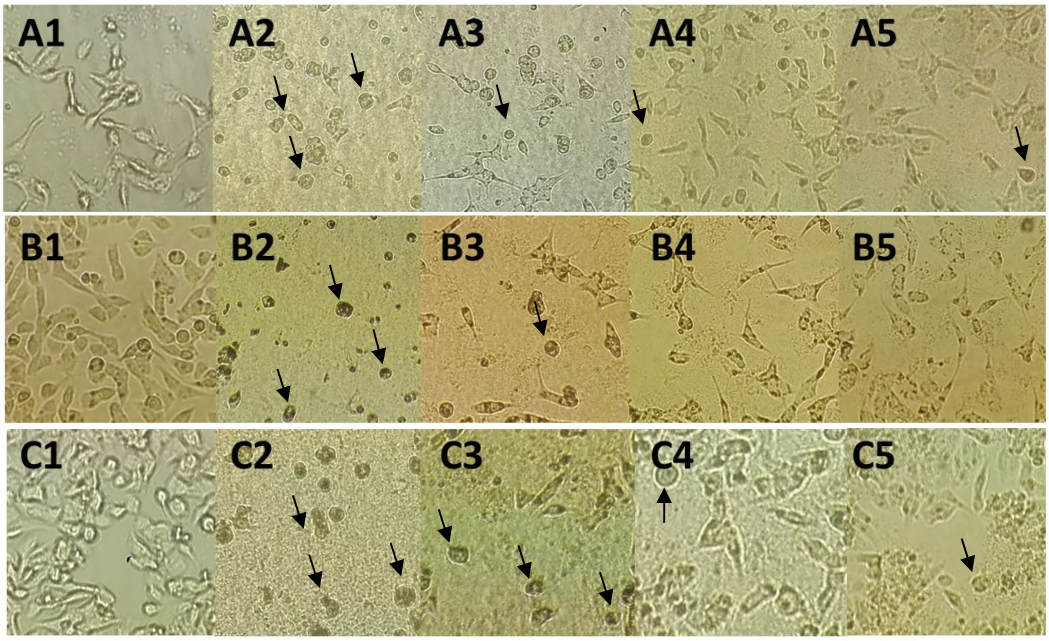

Figure S1. T47D cells were treated with Venom-loaded NPs with a resolution of 20x in a light microscope after A) 24h, B) 48h, and C) 72h. Concentrations used were (1) 0, (2) 250, (3) 125, (4) 62.5, and (5) 31.25 µg/mL. Morphological changes were indicated with arrows.

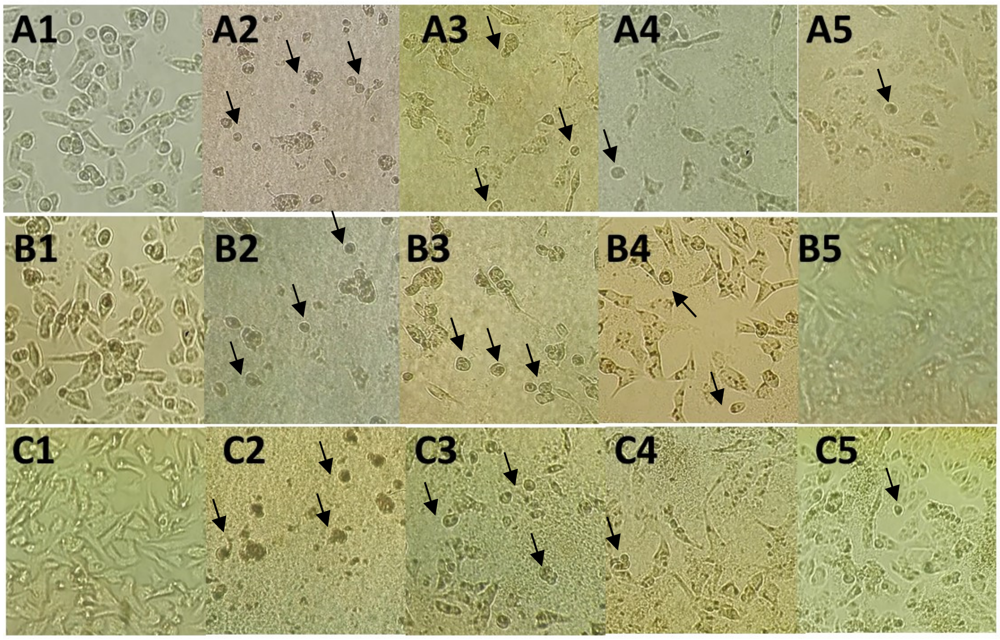

Figure S2. MDA-MB-231 cells were treated with Venom-loaded NPs with a resolution of 20x in a light microscope after A) 24h, B) 48h, and C) 72h. Concentrations used were (1) 0, (2) 250, (3) 125, (4) 62.5, and (5) 31.25 µg/mL. Morphological changes were indicated with arrows.

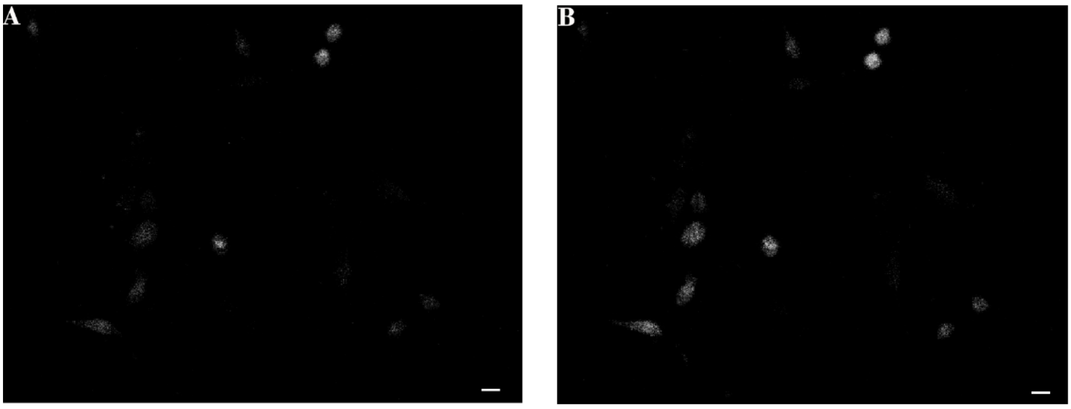

Figure S3. T47D cells loaded with 1 M Fluo 4 AM. Intracellular Ca^{2+} imaging of T47D cells superfused with Saline Solution (A, control) and during the C. atrox venom application (venom dissolved in saline solution, B). As illustrated in B, the intensity of Fluo 4 (arbitrary units) increased in all cells. This intracellular Ca^{2+} rise remained during the whole experiment (up to 12 min). These experiments represent n > 20 T47D cells.
